# Repairing of recurrent leg ulcer induced by hydroxyurea with posterior tibial artery perforator propeller flap: Case report

**DOI:** 10.1016/j.ijscr.2023.109049

**Published:** 2023-11-23

**Authors:** Zheng-Dong Wan, Wu-Zhou Li

**Affiliations:** a Department of Vascular and Endovascular Surgery, The First Affiliated Hospital of Yangtze University, Jingzhou 434000, Hubei, China; b Department of Plastic Surgery, The First Affiliated Hospital of Yangtze University, Jingzhou 434000, Hubei, China

**Keywords:** Leg ulcer, Essential thrombocythemia, Hydroxyurea, Posterior tibial artery, Perforator propeller flap, Case report

## Abstract

**Introduction and importance:**

Hydroxyurea is a cytotoxic drug commonly used to treat various myeloproliferative disorders. However, prolonged oral administration of this drug may trigger skin side effects and ulcers. There are few clinical reports on treating leg ulcers caused by hydroxyurea and even fewer clinical reports on managing recurrent ulcers after treatment.

**Case presentation:**

An 87-year-old woman with essential thrombocythemia presented with a painful skin ulcer on her left calf. After failed outpatient treatment, she opted for hospitalisation for free skin grafting. Four months later, ulcers reappeared at the transplant site, leading to her readmission to the hospital. The diagnosis revealed that the leg ulcers were caused by hydroxyurea. Despite this, she persisted with hydroxyurea treatment and subsequently underwent posterior tibial artery perforator flap surgery. During the two-year follow-up, a new ulcer developed on the medial condyle of her other calf. However, no new ulcers or local pain were observed in the area where perforator flap grafting was performed.

**Clinical discussion:**

Leg ulcers caused by hydroxyurea are rare clinically and can easily be misdiagnosed. There is currently minimal research on ulcer recurrence after treatment. Posterior tibial perforator flaps may more effectively promote the healing of recurrent ulcers.

**Conclusion:**

Compared to conservative treatment and skin grafting surgery, the posterior tibial artery perforator flap offers a dependable blood supply and enhances the likelihood of wound healing. It can be considered an option, particularly for recurrent and refractory ulcers, even without discontinuing medication.

## Highlights

• Recurrence of leg ulcers induced by hydroxyurea after treatment is rarely reported.
• The cure for ulcers without interrupting hydroxyurea treatment has not been reported.
• The repair of hydroxyurea-induced ulcers with PTA perforator flap is rarely reported.
• In this case, the ulcer healed well despite the patient still taking hydroxyurea.

## 1 Introduction

Hydroxyurea is a cytostatic agent that inhibits DNA synthesis by blocking ribonucleotide reductase. It has the characteristics of low toxicity and good tolerance. It is currently a commonly used drug for treating various myeloproliferative diseases. According to literature reports, long-term use of hydroxyurea can cause damage to organs such as the lungs [1], liver [2], and intestines [3]. The side effects of hydroxyurea on the skin can be manifested as dry skin, skin atrophy, rash, myositis-like skin lesions, and ulcers in severe cases [4,5]. The mechanism of hydroxyurea-induced ulcers is not yet precise and may be related to many factors [6].

Due to the lack of clinical reports, there are currently no guidelines for treating leg ulcers caused by hydroxyurea. Most reports suggested drug withdrawal or dose reduction, and the effect is specific. Sirieix et al. reported that about 80 % (33/41) of patients with leg ulcers caused by hydroxyurea could be wholly relieved after drug withdrawal [7]. A few case reports used skin graft therapy with unclear efficacy [8]. Clinical research is scarce on effectively managing ulcer recurrence following treatment, particularly in patients who do not intend to cease hydroxyurea therapy. Here, we report a successful repair case with a posterior tibial artery perforator flap.

This case report was prepared in accordance with the SCARE guidelines [9].

## 2 Case report

**Fig. 1:**
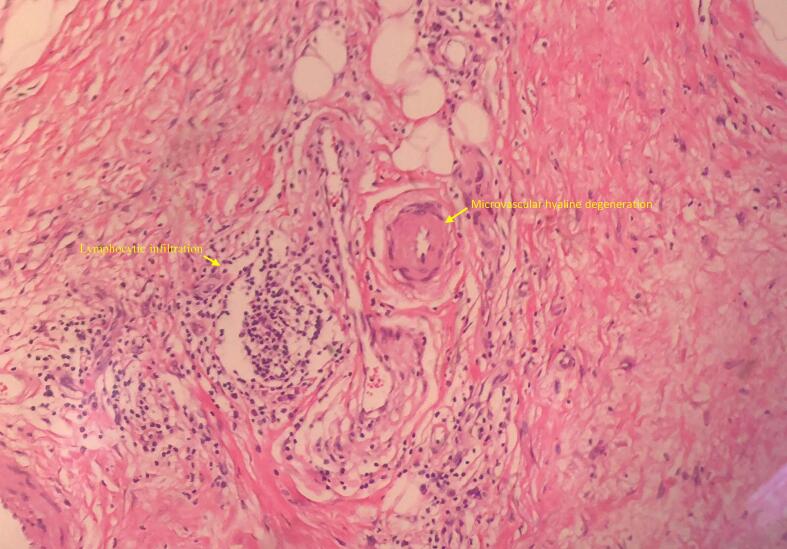
Histological section of the recurrent ulcer (HE, X 200).

**Fig. 2:**
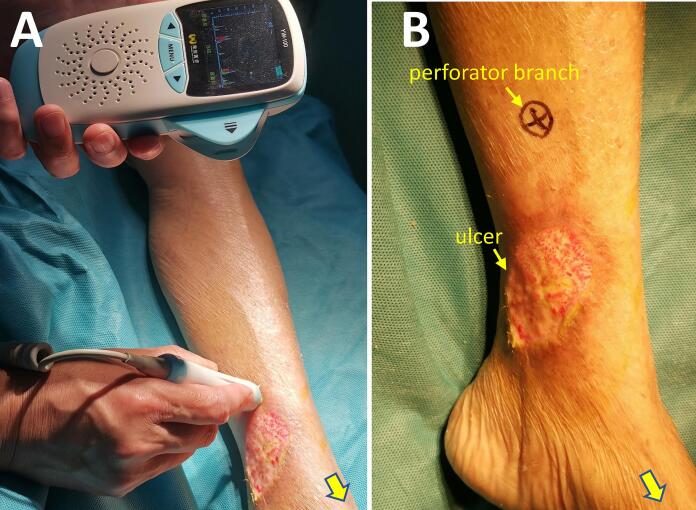
Selection and location of perforating branch of the left posterior tibial artery. A: Location of the perforator with the Doppler blood flow detector. B: the location of the selected perforator branch was marked on the skin. (The yellow arrow points to the direction of the foot.)

**Fig. 3:**
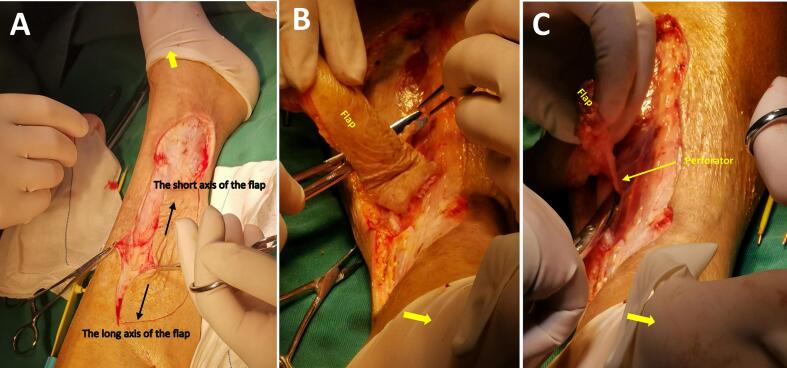
Design and preparation of posterior tibial artery perforator propeller flap. A: Design of the flap. B: Preparation of the flap. C: The flap was fully prepared. (The yellow arrow points to the direction of the foot.)

**Fig. 4:**
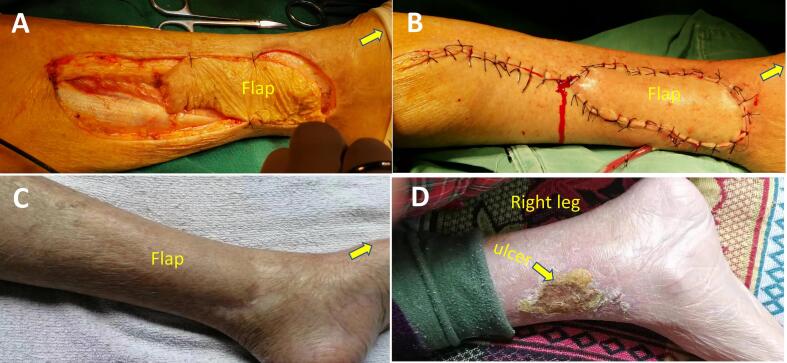
Wound closure and healing, follow up. A: The long axis of the flap fully covers the wound. B: The wound is fully sutured and drained. C: Wound appearance two years after the surgery. D: New ulcer at the opposite leg two years after surgery. (The yellow arrow points to the direction of the foot.)

An 87-year-old female patient presented with a one-month history of skin ulcer of the medial side of the left lower leg with unbearable local pain. Before being admitted to our hospital, she had two weeks of outpatient wound care in the outpatient department. The wound exudation was reduced, and local infection was controlled, but the wound still showed no signs of healing, and the local pain did not improve significantly. She had a medical history of essential thrombocytosis by bone marrow cytology in our hospital ten years ago. She was treated with oral hydroxyurea (Three times a day, 0.5 g each time.). She denied any history of trauma, diabetes, or allergy to particular drugs. Physical examination showed a skin ulcer above the medial ankle of the left leg, about 6 cm × 5 cm in size; no oedema or pigmentation was found in both lower extremities; bilateral dorsalis pedis arteries and posterior tibial arteries can be palpated. She was subsequently diagnosed with a possible venous ulcer and was advised to be hospitalised for an ultrasound examination. However, the bilateral great saphenous veins and deep vein systems of lower limbs showed normal findings. The arteries of both lower extremities were mild to moderately calcified, no apparent stenosis was found, and the blood flow velocities of the femoral artery, popliteal artery, and lower leg arteries were average. Laboratory examination: WBC: 13.60 × 109/L, PLT: 1096 × 109/L; Liver and kidney function, blood sugar, and lipids were normal. Considering the size of the ulcer, the wound is difficult to heal. She was advised to use a free medial up-thigh skin graft for skin grafting. After successful wound skin grafting, she recovered well and was discharged with the wound healed. However, four months after the discharge, the ulcer reoccurred at the same site. She has been admitted again. A wound biopsy revealed dermal microvascular hyaline degeneration with lymphocytic infiltration (Fig. 1). After multidisciplinary consultation, it was believed that the recurrent ulcer had a great relationship with the long-term use of hydroxyurea. Considering drug tolerability and family economic considerations, the patient decided to continue taking oral hydroxyurea treatment instead of discontinuing or switching medications. The possibility of ulcer unhealing was still high when free skin grafting was performed again. Considering that the repair of the flap with the vascular pedicle was more likely successful, an ultrasound examination of the right posterior tibial artery was performed, which showed that the diameter and position of the perforating branch met the surgical requirements. After communicating with the patient and the plastic surgeon, it was decided to use the posterior tibial artery perforator propeller flap to repair the ulcer. Before the operation, the perforating branch of the posterior tibial artery above the ulcer was detected with a Doppler blood flow detector (Fig. 2A). The perforating unit with the best blood flow signal was selected and marked (Fig. 2B). The operation was performed under epidural anaesthesia. Firstly, the ulcer and its base were resected along the 2 mm range around the ulcer. The flap was designed according to the size of the wound (Fig. 3A), the perforator vascular pedicle was freely mobilised (Fig. 3B, C), and the flap was rotated so that the long axis of the flap completely covered the resected ulcer area (Fig. 4A). Then, the flap and the edge of the incision were sutured with interrupted sutures. A drainage tube was placed under the wound (Fig. 4B). The wound healed well after the operation (Fig. 4C). After two years of follow-up, the patient developed a new ulcer on the contralateral calf over the medial condyle. No immediate treatment is required as the ulcer has already healed under the scab (Fig. 4D). It is worth mentioning that no new ulcers were observed at the site where the perforator flap graft was placed, and the patient did not report any local pain (Fig. 4C).

## 3 Discussion

A leg ulcer is a common clinical disease caused by various factors, including skin and soft tissue infection, trauma, lymphedema, deep vein thrombosis, lower extremity arterial ischemic disease, or venous reflux disorder [10]. Ulcers in the medial side of the lower leg are usually closely related to chronic venous insufficiency of the lower extremities, so they are also called venous ulcers and mainly occur in patients with varicose veins of the lower extremities and post-deep vein thromboembolism syndrome [11]. In our patient, the ulcer was located in the middle and lower part of the medial side of the left leg, which indicated a typical venous leg ulcer. However, we found no venous insufficiency of the lower extremity through an ultrasound examination of the extremity vein, so a venous ulcer was ruled out. Because the patient has a large-sized ulcer wound, choosing a wound skin graft is appropriate. However, ulcers recurred in the same area four months after the operation, suggesting that other related factors were not considered to affect the healing. After multidisciplinary discussion, it was considered related to long-term oral administration of hydroxyurea. Hydroxyurea, a hydroxylated urea derivative, inhibits cellular DNA synthesis by inhibiting ribonucleotide reductase [12]. It is the most commonly used chemotherapy drug in treating essential thrombocythemia. Long-term use can lead to intradermal vasculitis or microthrombosis, resulting in skin tissue damage and ulceration [13]. The biopsy results of the patient's wound revealed microvascular hyaline degeneration, potentially associated with the formation of microthrombi due to the patient's thrombocytosis and hydroxyurea treatment. This microcirculatory impairment can negatively impact wound healing and might contribute to the recurrence of ulcers following skin grafting in our patients. Hence, opting for vascularised flaps with superior healing capabilities would be more logical.

The posterior tibial artery perforator flap is commonly used in plastic surgery. Since there is no need to dissect the trunk of the posterior tibial artery, it does not affect the blood supply to the calf muscles and soft tissues [14]; the position of the perforator of the flap is relatively fixed, and the Doppler blood flow detector can be used to locate it before the operation accurately, and colour doppler ultrasound can also be used further to clarify the location and diameter of the perforator; the design of this flap is more flexible, and the appropriate flap size can be designed according to the ulcer area [15].

Due to the large ulcer area in our patient, the designed long-axis propeller flap was used to cover the wound well. At the same time, the blood perfusion of the flap was stable, and the wound healing ability was strong. The follow-up after two years showed that the flap's blood supply was good. Although the patient was still taking hydroxyurea orally, the ulcer did not recur.

## 4 Summary

In addition to common factors that trigger medial leg ulcers, it is essential to consider ulcers caused by drugs such as hydroxyurea [16]. The posterior tibial artery perforator flap is well-known for its reliable blood supply, which increases the chances of successful wound healing. Even if ongoing medical therapy has not been stopped, this flap can be considered a viable option for treating recurrent and refractory ulcers. However, it is crucial to recognise that patients who continue taking oral hydroxyurea are at an increased risk of early and late flap failure as well as new ulceration.

## Ethical approval

This case report has been reviewed and approved by the Medical Ethics Committee of the First Affiliated Hospital of Yangtze University, Ethics No. KY2023116.

## Sources of funding

None.

## CRediT authorship contribution statement

Zheng-Dong Wan and Wu-Zhou Li contributed to the data collection and manuscript writing.

## Guarantor

Zheng-Dong Wan.

## Research registration (for case reports detailing a new surgical technique or new equipment/technology)

None.

## Provenance and peer review

Not commissioned, externally peer-reviewed.

## Consent

Written informed consent was obtained from the patient for publication of this case report and accompanying images. A copy of the written consent is available for review by the Editor-in-Chief of this journal on request.

## Declaration of competing interest

None.
